# Development and validation of a nomogram for predicting cardiovascular mortality risk for diffuse large B-cell lymphoma in children, adolescents, and adults

**DOI:** 10.3389/fped.2024.1346006

**Published:** 2024-02-07

**Authors:** Kai Mu, Jing Zhang, Yan Gu, Guoying Huang

**Affiliations:** ^1^ Pediatric Heart Center, Children’s Hospital of Fudan University, Shanghai, China; ^2^ Department of Pediatric, The First Affiliated Hospital of Shandong First Medical University, Jinan, China

**Keywords:** pediatric cardiology, cardiovascular mortality, cardio-oncology, diffuse large B-cell lymphoma, nomogram

## Abstract

**Objective:**

This study aimed to construct and validate a nomogram for predicting cardiovascular mortality (CVM) for child, adolescent, and adult patients with diffuse large B-cell lymphoma (DLBCL).

**Materials and methods:**

Patients with only one primary tumor of DLBCL first diagnosed between 2000 and 2019 in the SEER database were extracted. We used the cumulative incidence function (CIF) to evaluate the cumulative rate of CVM. The outcome of interest was CVM, which was analyzed using a competing risk model, accounting for death due to other causes. The total database was randomly divided into a training cohort and an internal validation cohort at a ratio of 7:3. Adjustments were for demographics, tumor characteristics, and treatment modalities. Nomograms were constructed according to these risk factors to predict CVM risk at 5, 10, and 15 years. Validation included receiver operating characteristic (ROC) curves, time-dependent ROC, C-index, calibration curves, and decision curve analysis.

**Results:**

One hundred four thousand six hundred six patients following initial diagnosis of DLBCL were included (58.3% male, median age 64 years, range 0–80, White 83.98%). Among them, 5.02% died of CVM, with a median follow-up time of 61 (31–98) months. Nomograms based on the seven risk factors (age at diagnosis, gender, race, tumor grade, Ann Arbor stage, radiation, chemotherapy) with hazard ratios ranging from 0.19–1.17 showed excellent discrimination, and calibration plots demonstrated satisfactory prediction. The 5-, 10-, and 15-year AUC and C-index of CVM in the training set were 0.716 (0.714–0.718), 0.713 (0.711–0.715), 0.706 (0.704–0.708), 0.731, 0.727, and 0.719; the corresponding figures for the validation set were 0.705 (0.688–0.722), 0.704 (0.689–0.718), 0.707 (0.693–0.722), 0.698, 0.698, and 0.699. Decision curve analysis revealed a clinically beneficial net benefit.

**Conclusions:**

We first built the nomogram model for DLBCL patients with satisfactory prediction and excellent discrimination, which might play an essential role in helping physicians enact better treatment strategies at the time of initial diagnosis.

## Introduction

Diffuse large B-cell lymphoma (DLBCL) is the most common subtype of non-Hodgkin lymphoma (NHL) (1), making up roughly 40% of the total NHL population (2, 3). With improvements in treatment methods and increased survival rates, the number of DLBCL survivors is continually growing (2–4). The conventional treatment regimen for DLBCL commonly incorporates drugs like cyclophosphamide, doxorubicin, and rituximab, all of which carry a heightened risk of cardiac damage (1). Therefore, these survivors face an elevated risk of cardiovascular mortality (CVM) (5–7). While numerous previous studies (8–10) have discussed the incidence of cardiovascular events, there is limited literature addressing the CVM risk in DLBCL patients, especially in children and adolescents. To the best of our knowledge, there are no reported studies that have constructed and validated a nomogram for assessing the CVM risk in children, adolescents, and adults with DLBCL.

In child, adolescent, and adult DLBCL survivors, CVM might be related to disease stages, treatment modalities, and genetic factors (6, 7, 11). The challenges in estimating CVM risks have been highlighted in previous studies (5, 7, 12, 13). Previous studies (5, 14) have primarily utilized Cox regression models to investigate the risk of cardiovascular death and specifically focus on the elderly population. Our research distinctively employs a competing risks model for a more nuanced analysis. This approach incorporates the concept of competitive risks, mitigates the impact of death from other causes on the final outcomes, and includes individuals across the entire age spectrum, which facilitates the attainment of more reliable results (15, 16).

This study aims to construct and validate a nomogram to predict the risk of CVM for child, adolescent, and adult DLBCL patients. By better understanding these relationships, we can provide more targeted guidance for the long-term cardiovascular health management of DLBCL survivors, particularly for the younger age demographic, thereby improving their quality of life and survival prospects.

## Materials and methods

### Data source and study population

As a network of U.S. population-based incident tumor registries, the Surveillance, Epidemiology, and End Results (SEER) program, which is a public registry maintained by the National Cancer Institute, currently encompasses approximately 27.8% of the cancer patient population (17). The SEER collects patient clinical information data, such as demographics, primary tumor site, stage at diagnosis, initial course of treatment, follow-up time, survival, and economic status of residence. We extracted data from patients with only one primary DLBCL between 2000 and 2019 in the SEER database using the SEER*Stat software (version 8.4.2).

**Figure 1:**
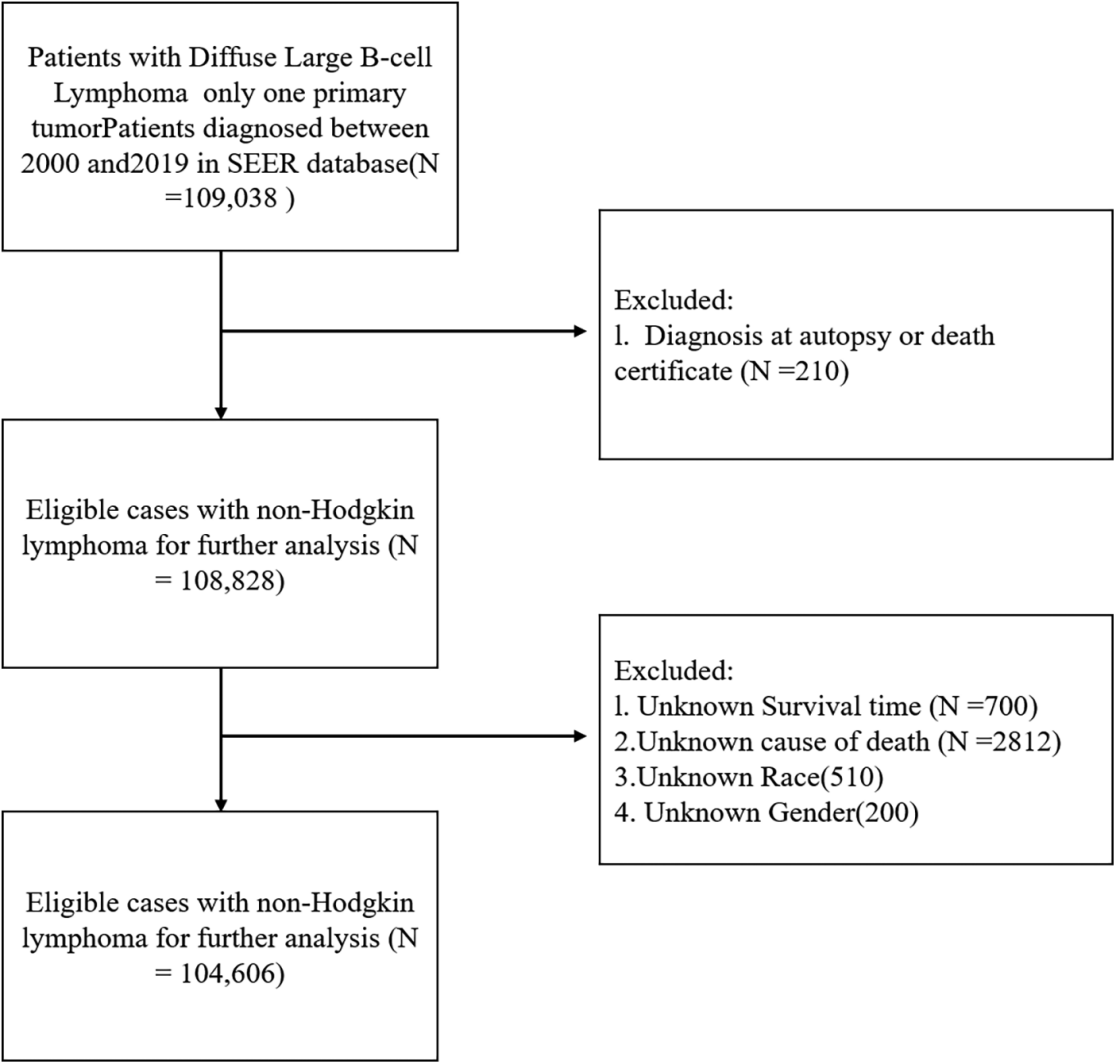
Flowchart depicting the enrolment of patients based on inclusion and exclusion criteria.

We utilized histology codes from the third edition of the International Classification of Diseases for Oncology (ICD-O-3)—codes 9680/3, 9684/3, and 9688/3—to assemble the cohort of interest, including children, adolescents, and adults aged 0–80 years following initial diagnosis of DLBCL. Patients with a diagnosis at autopsy or death certificate and those with incomplete data on certain variables (survival time, cause of death, race, gender) were excluded (Figure 1).

### Risk factors

The variables included in this study are based on clinical support (18, 19) and include age at diagnosis (0–18 years, 19–40 years, 41–60 years, 61–80 years), gender, year of diagnosis, tumor grade (no nodal or metastatic disease as local, nodal disease as regional, or any metastatic disease as distant), race (white, black, or other), cause of death, survival time, the Ann Arbor stage (Stage I: Early-stage cancer confined to the organ of origin. Stage II: Local spread, potentially involving nearby lymph nodes. Stage III: More extensive local spread, involving additional lymph nodes. Stage IV: Presence of distant metastasis.), primary site, mean household income (<$60,000, >$60,000), surgery (yes, no), chemotherapy (yes, no/unknown), radiotherapy (yes, no/unknown), and place of residence (rural, urban). Primary site codes were used from SEER*Stat for the “lip, oral, cavity and pharynx” (C0.0–14.9), digestive organs (C15.0–C27.0), “respiratory and intrathoracic organs” (C30.0–C39.9), “bone, joints and articular cartilage” (C40.0–C41.9), hematopoietic (C42.0–C42.9), skin (C44.0–C44.9), nervous system (C47.0–C47.9), peritoneum (C48.0–C48.9), soft tissues (C49.0–C49.9), breast (C50.0–C50.9), female genital organs (C51.0–C57.9), male genital organs (C60.0–C63.9), urinary tract (C64.0–C68.9), “eye, brain and other parts of central nervous system” (C69.0–C72.9), “endocrine system” (C73.0–C76.9), “lymph nodes” (C77.0–C78.0). County median income level was dichotomized into groups based on the SEER-linked county-level data regarding the median household income in the past 12 months using 2019 inflation-adjusted dollars. The initial course of treatment was determined based on whether patients received surgery, chemotherapy, or radiation therapy. Survival time refers to the interval from the diagnosis of cancer to the death of patients due to any cause or the last date of available survival information.

### Outcomes

The main outcome of interest was a composite of CVM, defined as any of the following seven causes of death in the SEER database [International Classification of Diseases, 10th Revision (ICD-10) codes]: heart diseases (I00–I09, I11, I13, I20–I51), hypertension without heart disease (I10, I12), cerebrovascular diseases (I60–I69), atherosclerosis (I70), aortic aneurysm and dissection (I71), and other diseases of arteries, arterioles, and capillaries (I72–I78). Competitive risk refers to mortality due to other causes (such as primary diffuse large B-cell lymphoma, infection, and bleeding).

### Statistical analysis

For calculating the sample size required for developing a clinical prediction model, the sample size calculation satisfied both the 15 EPV (events per variable) and pmsampsize rule requirements (20) (T1: estimate the overall outcome proportion with sufficient precision at one or more key time-points in follow-up; T2: target a shrinkage factor of 0.9; T3: target small optimism of 0.05 in the apparent R2Nagelkerke) in the training dataset. The R package “pmsampsize” was used, referring to some statistical parameters from a previous study (5). The minimum sample size calculated by the “pmsampsize” package was 1,450 cases. The sample size of the training dataset satisfies this requirement.

Normally distributed data were expressed as mean ± standard deviation. Non-normally distributed data were expressed as medians with interquartile ranges. Categorical data were presented using counts with percentages and compared using the chi-square test, while when frequencies were below 5, Fisher's exact test was applied.

In the competing risk analyses, we used the cumulative incidence function (CIF) to evaluate the cumulative rate of CVM. Multivariable competing risk survival analyses were performed to identify predictors of CVM. The total database was randomly divided into a training cohort and an internal validation cohort at a ratio of 7:3. The training cohort was used for risk factor analysis and nomogram construction. Factors with *p*-values < 0.05 in univariate competing risk analysis were added to a multivariate competing risk model to detect indicators of death specifically due to CVM. Based on the results of the competing risk analysis of the training cohort, a nomogram was constructed that incorporated all the independent prognostic factors to predict 5-, 10-, and 15-year CVM risk.

We evaluated the accuracy of the nomogram model by examining the 5-, 10-, and 15-year ROC curves. C-index for 5, 10, and 15 years were calculated to measure the predictive ability and accuracy of the model. The predicting outcomes of the nomogram were evaluated in the respective training and validation cohorts by calibrating curves and the decision curve analysis (DCA). DCA is a critical method for assessing the clinical utility of clinical predictive models and can address the limitations of the ROC curve (21).

Ethical approval of this publicly available information provided by the SEER program was not required by our Institutional Review Board. The manuscript has been prepared in accordance with the guideline of Transparent Reporting of a Multivariable Prediction Model for Individual Prognosis or Diagnosis (TRIPOD) (22). For detailed information regarding the TRIPOD checklist, please refer to the online supplementary appendix. Statistical analyses were performed using STATA-MP 17.0 (StataCorp, College Station, TX) and R software (version 4.1.2; R Foundation for Statistical Computing, Vienna, Austria). All statistical tests were two-sided, with the significance level set at a *p*-value of < 0.05.

## Results

### Patient characteristics

A total of 104,606 DLBCL were included in subsequent analyses. The median age at diagnosis was 64 (53.72) years. The proportion of male patients was 60,953 (58.27%) and the median follow-up time was 61 (31–98) months. The majority of patients were White 87,845 (83.98%) and 56,080 (53.61%) had distant tumor stage. The most common primary site was lymph nodes (64.46%), followed by the digestive system (9.34%) and nervous system (4.67%). A total of 27,713 (26.49%) patients underwent surgery, 82,825 (79.18%) patients received chemotherapy, and only 18,755 (17.93%) patients underwent radiotherapy. Furthermore, 5,254 (5.02%) patients died of CVD, 4,202 (4.02%) died due to heart diseases, 715 (0.68%) due to cerebrovascular diseases, 53 (0.05%) due to aortic aneurysm and dissection, 180 (0.17%) due to hypertension without heart disease, and 38 (0.04%) due to atherosclerosis. The baseline characteristics are detailed in Tables 1, 2.

**Table 1:** Clinicopathologic characteristics of patients in different Age groups.

	Levels	Total (*N* = 104,606)	0–18 (*N* = 1,026)	19–40 (*N* = 8,793)	41–60 (*N* = 32,373)	61–80 (*N* = 62,414)
Age at diagnosis		64.00 (53.00, 72.00)	15.00 (11.00, 17.00)	33.00 (28.00, 37.00)	53.00 (48.00, 57.00)	71.00 (66.00, 76.00)
Gender	Female	43,653 (41.73%)	383 (37.3%)	3,396 (38.6%)	12,212 (37.7%)	27,662 (44.3%)
	Male	60,953 (58.27%)	643 (62.7%)	5,397 (61.4%)	20,161 (62.3%)	34,752 (55.7%)
Race	Black	7,765 (7.42%)	142 (13.8%)	1,369 (15.6%)	3,291 (10.2%)	2,963 (4.7%)
	Other	8,996 (8.60%)	96 (9.4%)	897 (10.2%)	2,775 (8.6%)	5,228 (8.4%)
	White	87,845 (83.98%)	788 (76.8%)	6,527 (74.2%)	26,307 (81.3%)	54,223 (86.9%)
Time from diagnosis to therapy		61.00 (31.00–98.00)	91.00 (32.00–162.00)	75.00 (18.00–148.00)	55.00 (13.00–122.00)	28.00 (6.00–78.00)
Tumor grade	Distant	56,080 (53.61%)	422 (41.1%)	3,892 (44.3%)	17,491 (54%)	34,275 (54.9%)
	Localized	25,118 (24.01%)	335 (32.7%)	2,527 (28.7%)	7,615 (23.5%)	14,641 (23.5%)
	Regional	18,277 (17.47%)	225 (21.9%)	2,078 (23.6%)	5,835 (18%)	10,139 (16.2%)
	Unknown	5,131 (4.91%)	44 (4.3%)	296 (3.4%)	1,432 (4.4%)	3,359 (5.4%)
Ann Arbor stage	I	17,605 (16.83%)	226 (22%)	1,732 (19.7%)	5,368 (16.6%)	10,279 (16.5%)
	II	13,034 (12.46%)	158 (15.4%)	1,452 (16.5%)	4,215 (13%)	7,209 (11.6%)
	III	5,724 (5.47%)	53 (5.2%)	318 (3.6%)	1,831 (5.7%)	3,522 (5.6%)
	IV	13,023 (12.45%)	81 (7.9%)	809 (9.2%)	4,095 (12.6%)	8,038 (12.9%)
	Unknown	55,220 (52.79%)	508 (49.5%)	4,482 (51%)	16,864 (52.1%)	33,366 (53.5%)
Primary sit	Bones and joints	1,870 (1.79%)	94 (9.2%)	349 (4%)	503 (1.6%)	924 (1.5%)
	Brain and other nervous system	4,888 (4.67%)	34 (3.3%)	398 (4.5%)	1,514 (4.7%)	2,942 (4.7%)
	breast	768 (0.73%)	3 (0.3%)	55 (0.6%)	245 (0.8%)	465 (0.7%)
	Digestive system	9,765 (9.34%)	99 (9.6%)	658 (7.5%)	3,024 (9.3%)	5,984 (9.6%)
	Endocrine system	1,167 (1.12%)	4 (0.4%)	58 (0.7%)	357 (1.1%)	748 (1.2%)
	Eye and orbit	509 (0.49%)	4 (0.4%)	28 (0.3%)	142 (0.4%)	335 (0.5%)
	Female genital system	393 (0.38%)	1 (0.1%)	88 (1%)	153 (0.5%)	151 (0.2%)
	Hematopoietic	5,147 (4.92%)	13 (1.3%)	166 (1.9%)	1,499 (4.6%)	3,469 (5.6%)
	Lymph nodes	67,434 (64.46%)	606 (59.1%)	5,779 (65.7%)	21,155 (65.3%)	39,894 (63.9%)
	Male genital system	1,215 (1.16%)	4 (0.4%)	48 (0.5%)	289 (0.9%)	874 (1.4%)
	Oral cavity and pharynx	4,292 (4.10%)	71 (6.9%)	390 (4.4%)	1,485 (4.6%)	2,346 (3.8%)
	Peritoneum	372 (0.36%)	3 (0.3%)	20 (0.2%)	94 (0.3%)	255 (0.4%)
	Respiratory system	3,156 (3.02%)	56 (5.5%)	528 (6%)	891 (2.8%)	1,681 (2.7%)
	Skin	1,225 (1.17%)	6 (0.6%)	73 (0.8%)	365 (1.1%)	781 (1.3%)
	Soft tissue including heart	1,414 (1.35%)	13 (1.3%)	100 (1.1%)	407 (1.3%)	894 (1.4%)
	Unknown	414 (0.40%)	6 (0.6%)	19 (0.2%)	114 (0.4%)	275 (0.4%)
	Urinary system	577 (0.55%)	9 (0.9%)	36 (0.4%)	136 (0.4%)	396 (0.6%)
Radiation	None/unknown	85,851 (82.07%)	940 (91.6%)	6,397 (72.8%)	26,029 (80.4%)	52,485 (84.1%)
	Yes	18,755 (17.93%)	86 (8.4%)	2,396 (27.2%)	6,344 (19.6%)	9,929 (15.9%)
Chemotherapy	No/unknown	21,781 (20.82%)	100 (9.7%)	1,166 (13.3%)	5,491 (17%)	15,024 (24.1%)
	Yes	82,825 (79.18%)	926 (90.3%)	7,627 (86.7%)	26,882 (83%)	47,390 (75.9%)
Surgery	No	76,378 (73.01%)	702 (68.4%)	6,358 (72.3%)	23,003 (71.1%)	46,315 (74.2%)
	Unknown	515 (0.49%)	4 (0.4%)	41 (0.5%)	147 (0.5%)	323 (0.5%)
	Yes	27,713 (26.49%)	320 (31.2%)	2,394 (27.2%)	9,223 (28.5%)	15,776 (25.3%)
Year of diagnosis	2000–2004	21,724 (20.77%)	260 (25.3%)	2,389 (27.2%)	7,136 (22%)	11,939 (19.1%)
	2005–2009	24,043 (22.98%)	244 (23.8%)	2,190 (24.9%)	7,993 (24.7%)	13,616 (21.8%)
	2010–2014	27,952 (26.72%)	269 (26.2%)	2,107 (24%)	8,757 (27.1%)	16,819 (26.9%)
	2015–2019	30,887 (29.53%)	253 (24.7%)	2,107 (24%)	8,487 (26.2%)	20,040 (32.1%)
Income	<$60,000	28,306 (27.06%)	759 (74%)	6,711 (76.3%)	24,173 (74.7%)	44,642 (71.5%)
	>$60,000	76,285 (72.93%)	267 (26%)	2,081 (23.7%)	8,196 (25.3%)	17,762 (28.5%)
	Unknown	15 (0.01%)	0 (0%)	1 (0%)	4 (0%)	10 (0%)
Rural/urban	Rural	12,454 (11.91%)	84 (8.2%)	707 (8%)	3,392 (10.5%)	8,271 (13.3%)
	Unknown	91 (0.09%)	2 (0.2%)	7 (0.1%)	31 (0.1%)	51 (0.1%)
	Urban	92,061 (88.01%)	940 (91.6%)	8,079 (91.9%)	28,950 (89.4%)	54,092 (86.7%)

**Table 2:** Categorized results of DLBCL patients who died from cardiovascular diseases.

	Patients died from CV diseases (*N* = 5,254)
Heart diseases	4,202 (79.97%)
Hypertension without heart disease	180 (3.43%)
Cerebrovascular diseases	715 (13.61%)
Aortic aneurysm and dissection	53 (1.01%)
Atherosclerosis	38 (0.72%)
Other diseases of arteries, arterioles, capillaries	66 (1.26%)

### Univariate and multivariable analyses on the cardiovascular mortality

Risk factor analyses were based on the training set of 73,224 patients. The results of the univariate analysis are summarized in Table 3. In the univariate analysis, factors including age at diagnosis, gender, race, tumor grade, Ann Arbor stage, chemotherapy, and radiation were found to be associated with the CVM risk in child, adolescent, and adult patients with DLBCL.

**Table 3:** Effect of univariate competing risk result on the cardiovascular mortality.

	HR	(95% CI)	*P*
Age			
0–18 vs. 61–80	0.17	(0.14–0.21)	<0.001
19–40 vs. 61–80	0.32	(0.30–0.34)	<0.001
41–60 vs. 61–80	0.56	(0.54–0.57)	<0.001
Gender			
Male vs. female	1.13	(1.11–1.16)	<0.001
Race			
Other* vs. black	0.85	(0.78–0.89)	<0.001
White vs. black	0.87	(0.83–0.91)	<0.001
Ann Arbor stage			
Stage II vs. stage I	0.94	(0.90–0.99)	<0.001
Stage III vs. stage I	1.30	(1.23–1.38)	<0.001
Stage IV vs. stage I	1.71	(1.64–1.78)	<0.001
Unknown vs. stage I	1.57	(1.52–1.62)	<0.001
Tumor grade			
Localized vs. distant	0.60	(0.59–0.62)	<0.001
Regional vs. distant	0.59	(0.57–0.61)	<0.001
Unknown vs. distant	0.91	(0.87–0.96)	<0.001
Radiation			
Yes vs. no	0.64	(0.62–0.66)	<0.001
Chemotherapy			
Yes vs. no	0.54	(0.53–0.55)	<0.001
Surgery			
Yes vs. no	0.82	(0.80–0.84)	<0.001
Unknown vs. no	1.14	(0.98–1.32)	0.071
Rural/urban			
Urban vs. rural	0.87	(0.84–0.89)	<0.001
Unknown vs. rural	1.11	(0.77–1.58)	0.566

### Risk factor analysis

The multivariate analysis of CVM conducted by competing risk analysis in the training cohort is summarized in Table 4. Multivariate analysis revealed that the following characteristics were associated with risk of CVM: age at diagnosis ([0–18 vs. 61–80] HR: 0.19, 95% CI: 0.15–0.23; [19–40 vs. 61–80] HR: 0.34, 95% CI: 0.32–0.36; [41–60 vs. 61–80] HR: 0.57, 95% CI: 0.55–0.58), gender [(Male vs. Female) HR: 1.17, 95% CI: 1.15–1.21], race [(White vs. Black) HR: 0.73, 95% CI:0.69–0.76], tumor grade ([Localized vs. Distant] HR: 0.62, 95% CI: 0.58–0.65; [Regional vs. Distant] HR: 0.72, 95% CI: 0.68–0.76), Ann Arbor stage ([Stage II vs. Stage I] HR: 0.89, 95% CI: 0.82–0.96; [Stage III vs. Stage I] HR: 0.82, 95% CI: 0.76–0.89; [Stage IV vs. Stage I] HR: 1.05, 95% CI: 0.98–1.11). In terms of treatment, radiation [(Yes vs. No) HR: 0.85, 95% CI: 0.82–0.88] and chemotherapy [(Yes vs. No) HR: 0.56, 95% CI: 0.55–0.57] were associated with CVM in DLBCL patients.

**Table 4:** Multivariate competing risk result on the cardiovascular mortality.

	HR	(95% CI)	*P*
Age			
0–18 vs. 61–80	0.19	(0.15–0.23)	<0.001
19–40 vs. 61–80	0.34	(0.32–0.36)	<0.001
41–60 vs. 61–80	0.57	(0.55–0.58)	<0.001
Gender			
Male vs. female	1.17	(1.15–1.21)	<0.001
Race			
Other vs. black	0.78	(0.73–0.82)	<0.001
White vs. black	0.73	(0.69–0.76)	<0.001
Ann Arbor stage			
Stage II vs. stage I	0.89	(0.82–0.96)	<0.001
Stage III vs. stage I	0.82	(0.76–0.89)	0.005
Stage IV vs. stage I	1.05	(0.98–1.11)	<0.001
Unknown vs. stage I	1.09	(1.04–1.16)	<0.001
Tumor grade			
Localized vs. distant	0.62	(0.58–0.65)	<0.001
Regional vs. distant	0.72	(0.68–0.76)	<0.001
Unknown vs. distant	0.67	(0.64–0.71)	<0.001
Radiation			
Yes vs. no	0.85	(0.82–0.88)	<0.001
Chemotherapy			
Yes vs. no	0.56	(0.55–0.57)	<0.001

### Developing nomograms

**Figure 2:**
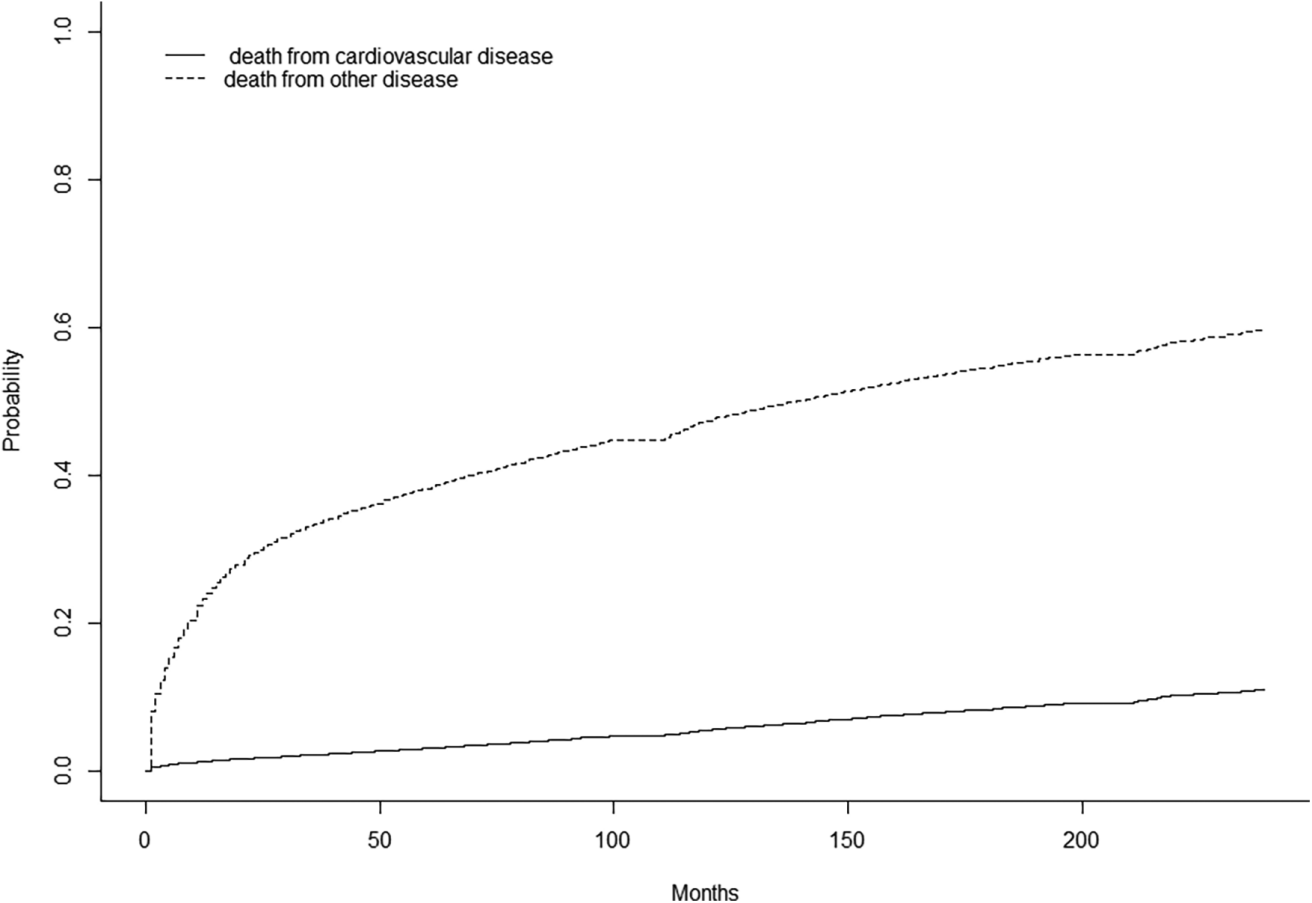
Cumulative mortality for all causes of death in DLBCL patients.

The results of the CIF curves of cause of death in DLBCL patients are illustrated in Figure 2 and show that CVM increases with survival time.

**Figure 3:**
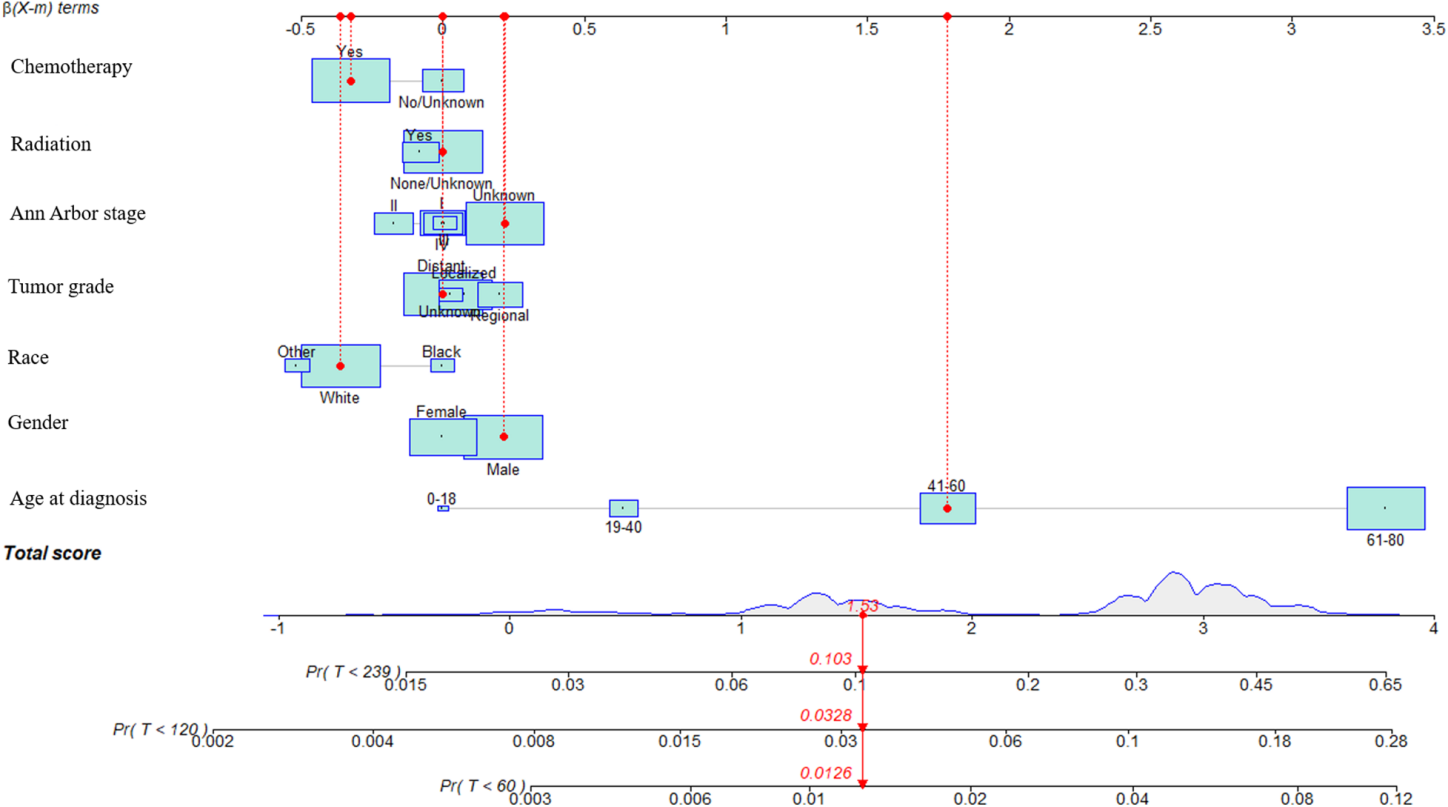
Nomograms to predict CVM for patients with DLBCL and risk stratification. In this nomogram, each variable corresponds to a point on the axis of the nomogram, and the corresponding score of the variable was obtained. The sum score of each variable was obtained, the total score corresponded to the point on the risk axis, and the risk value of CVM was obtained.

In the training cohort, prognostic factors were used to construct the nomogram of CVM for predicting 5-, 10-, and 15-year CVM risk, as presented in Figure 3. In this nomogram, each variable corresponds to a point on the axis of the nomogram, and the corresponding score of the variable was obtained. The sum score of each variable was obtained, the total score corresponded to the point on the risk axis, and the risk value of CVM was obtained.

### Internal validation

**Figure 4:**
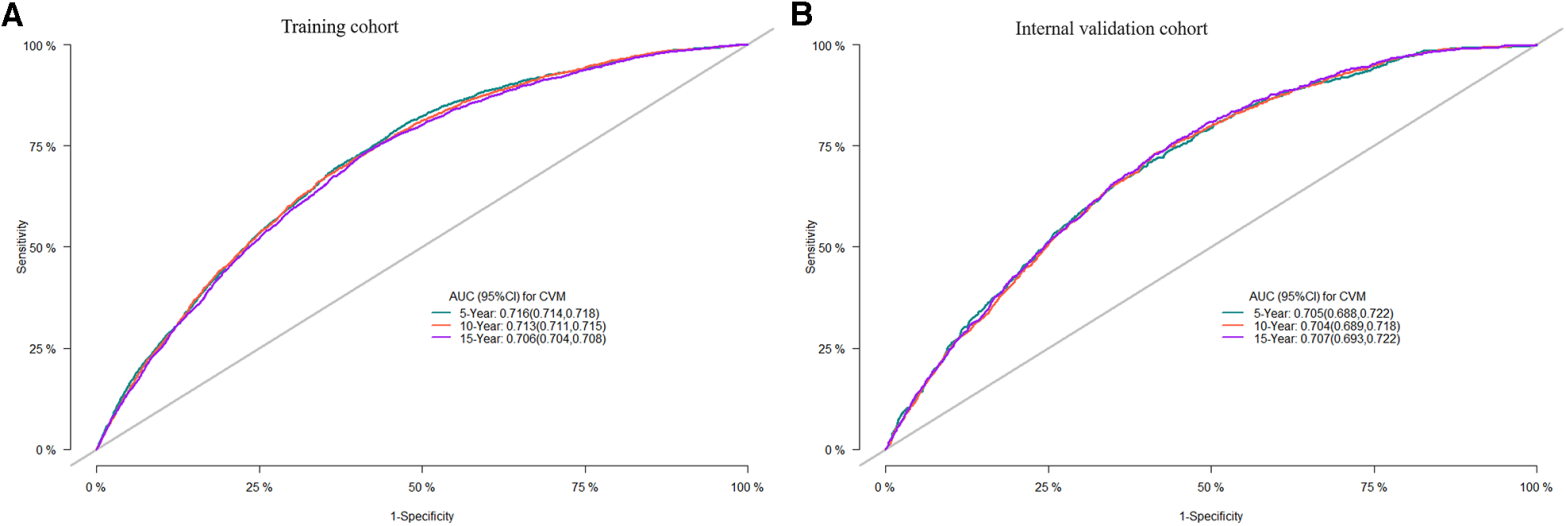
ROC curves of the nomogram of DLBCL patients for 5-, 10-, and 15-year CVM rates in the training cohort (**A**), the internal validation cohort (**B**); ROC, receiver operating characteristic; AUC, area under the curve.

**Figure 5:**
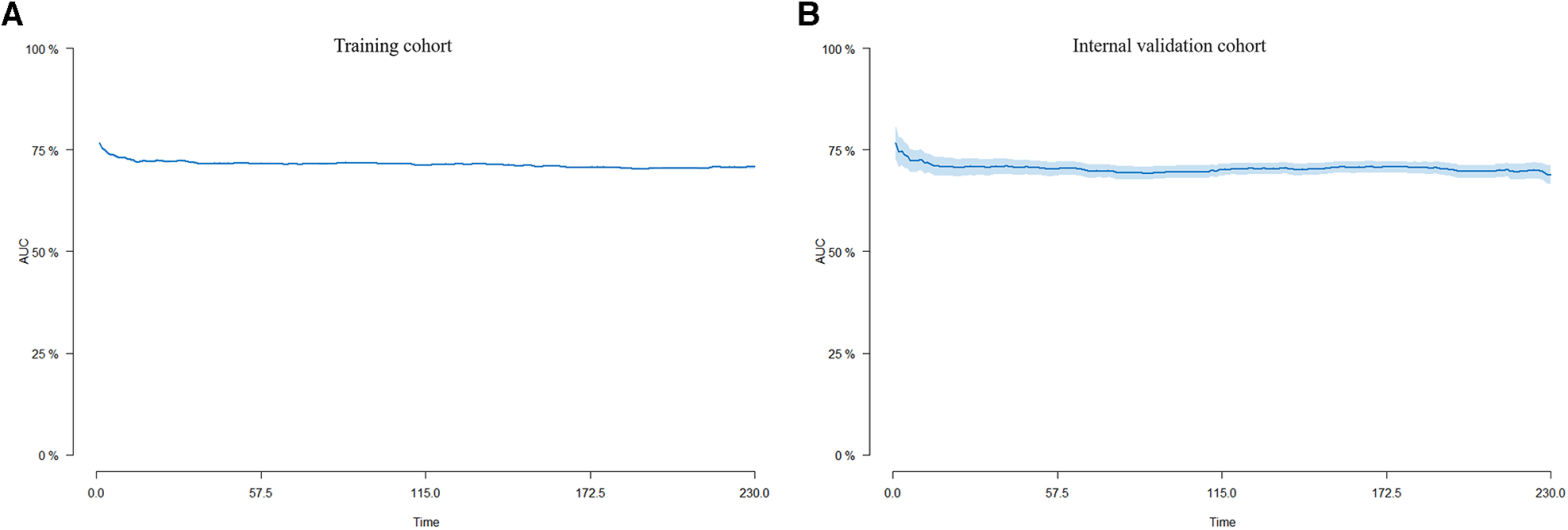
Time-dependent AUC curves for the nomogram of CVM in the training cohort (**A**) and the internal validation cohort (**B**).

**Figure 6:**
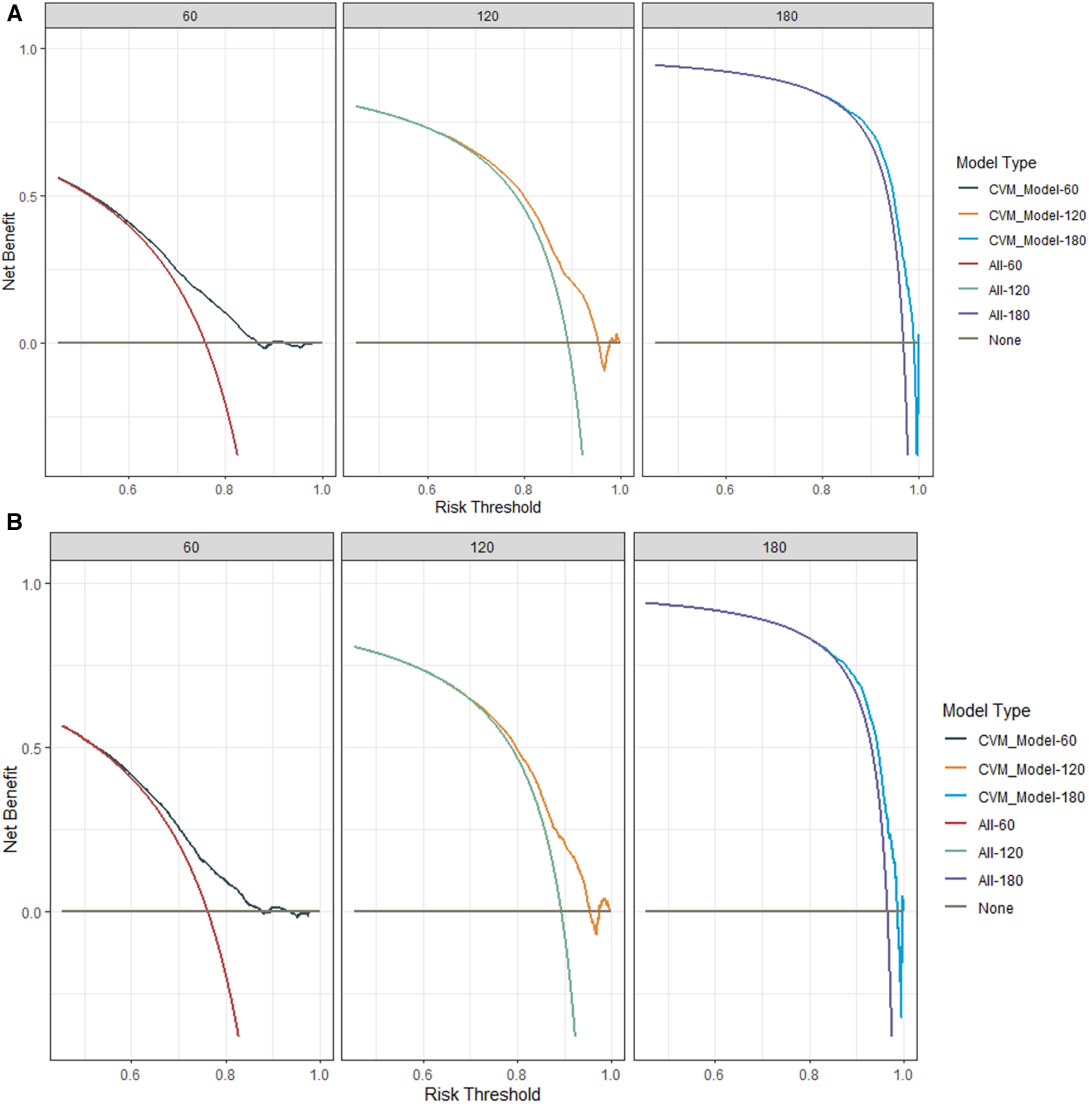
The decision-curve analysis (DCA) plots of the nomogram of DLBCL patients for 60-, 120-, and 180-month CVM rates in the training cohort (**A**) and the internal validation cohort (**B**).

Internal validation was performed in a cohort of 31,382 patients. Patient baseline characteristics between the two cohorts were well-balanced (Supplementary Table S1). Validation methods demonstrated efficacy and stability. The 5-, 10-, and 15-year ROC curves of CVM and time-dependent ROC curves of the CVM nomogram were displayed (Figures 4, 5). The 5-, 10-, and 15-year AUC of CVM in the training set were 0.716 (0.714–0.718), 0.713 (0.711–0.715), and 0.706 (0.704–0.708), with C-index 0.731, 0.727, and 0.719, respectively; the corroding figures for the validation set were 0.705 (0.688–0.722), 0.704 (0.689–0.718), and 0.707 (0.693–0.722), with C-index 0.698, 0.698, and 0.699, respectively (Table 5). The calibration curves were very close to the diagonal (Supplementary Figure S1) showed high consistencies between the predicted and observed CVM probability in the cohorts. This demonstrates good predictive ability and accuracy of the model. The decision curve analysis for the model is shown in Figure 6. The X-axis indicates the threshold probability for CVM, while the Y-axis indicates the net benefit. The DCA indicated a significantly better net benefit, indicating the effective use of the model in achieving net clinical benefit.

**Table 5:** C-index for nomogram model.

Cohort	C-index		
	5-year	10-year	15-year
Training-cohort	0.731	0.727	0.719
Validation cohort	0.698	0.698	0.699

## Discussion

Utilizing the SEER database of more than 10,000 patients first diagnosed with DLBCL between the ages of 0 and 80 years, we built the first nomogram to predict the risk of CVM, with excellent results in internal validation. This research reports a 5.02% cumulative occurrence rate of cardiovascular death in diffuse large B-cell lymphoma patients, identifying significant risk factors for predicting CVM, including age at diagnosis, gender, tumor grade, Ann Arbor stage, radiation, and chemotherapy. The reported AUC exceeds 0.70, indicating good predictive ability. The risk of CVM decreases with earlier diagnosis, as segmented by age groups.

DLBCL is a type of non-Hodgkin's lymphoma, of which complications such as cardiomyopathy and rhythm disturbances are frequently documented in children, adolescents, and adults (3, 12). In most studies (8, 23), the prevalence of cardiovascular disease is described rather than the cardiovascular disease mortality rate. In our current study, we observed a CVM rate of 5.02% among DLBCL patients. This finding is corroborated by the work of Jurczak et al. (24), who reported a 4.59% rate of cardiovascular-related deaths in DLBCL patients undergoing treatment. Additionally, our research demonstrates that for diffuse large B-cell lymphoma patients, an early diagnosis in childhood correlates with a reduced risk of cardiovascular death. A recent study disclosed that among the causes of cardiovascular death, heart-related conditions were the most predominant, accounting for 79.4% of cases, followed by cerebrovascular diseases, at 14.4% (5). These findings are consistent with our own results.

Our findings emphasize that age at diagnosis has a notable correlation with the risk of CVM, in line with previous studies (25–27). This association is particularly pronounced among older individuals, who frequently present with an advanced stage of the disease. Additionally, factors such as limited tolerance to adequate chemotherapy and the presence of pre-existing comorbidities, especially cardiac conditions, exacerbate the risk within this demographic (28). The heightened treatment toxicity observed in elderly patients is further influenced by the diminished reserve capacity of organs susceptible to these toxic effects.

When comparing gender, we observed a higher risk among men than among women. This is consistent with the findings reported in other literature (5, 27, 29–31). This may be attributed to men's metabolism of chemotherapy drugs, which leads to a lower response and survival (32). Race was also identified as a predictor of CVM in our study. This is consistent with the findings of previous studies (27, 30), which reported that black patients had a higher risk of CVM compared to white DLBCL patients. This could be due to disparities in access to healthcare and treatment.

The tumor grade was also identified as a significant predictor of CVM in our study. The findings from the study conducted by Kamel et al. (5) are in alignment with our observations, revealing that patients with a high tumor grade are at an elevated risk of CVM. This could be due to the aggressive nature of high-tumor grade, which often requires intensive treatment that can increase the risk of cardiovascular disease. Simultaneously, in our research, our study revealed that the Ann Arbor stage was correlated with the risk of CVM. This result has also been observed in other research articles (5, 33). Additional clinical research is required to elucidate the predictive roles of tumor grade and Ann Arbor stage in assessing the risk of CVM among DLBCL patients.

Radiotherapy emerged as the pioneering treatment, offering enduring remission and even a potential cure for DLBCL patients (34, 35). Contrary to traditional understanding, mediastinal or thoracic region radiation can inflict considerable harm on the heart and adjacent vasculature, thereby elevating the risk of ischemic heart disease and valvular irregularities. There is controversy around whether radiotherapy is appropriate for DLBCL patients. Additionally, in our study, both univariate and multivariate competing risk models indicated that patients who underwent radiation therapy had lower cardiovascular disease-related mortality compared to those who did not receive radiation treatment. Our findings are consistent with the studies (35, 36), indicating that the mortality rate is minimized through exposure to radiotherapy compared to patients who did not receive radiation. The latter group showed a higher risk of mortality and a lower survival rate. This may suggest that undergoing radiation therapy is associated with reduced cardiovascular-related mortality rates. Zimmermann et al. (37) indicate that the omission of radiation leads not only to a shorter progression-free survival (PFS) but also to worse overall survival (OS). Radiation should therefore remain standard practice. In the SEER program, a detailed radiotherapy regimen was lacking. As such, additional research is necessary to elucidate the impact of radiotherapy on the risk of CVM in patients diagnosed with DLBCL.

### Strengths and limitations

The current study offers several notable advantages that contribute to both its credibility and broader applicability. To begin with, our study has verified the risk factors for CVM in children, adolescents, and adults with DLBL and has constructed the first predictive model for all age groups to predict CVM. However, our study has some limitations. First, the subjects included in our study were patients with initial, solitary occurrences of diffuse large B-cell lymphoma, representing a selective approach. Second, some information associated with CVM that might influence survival was not available, such as doses of radiation, chemotherapy regimens, smoking, alcohol use, transcriptomic or genomic data, and history of cardiovascular diseases. Third, because our study patients were predominantly White, the generalizability of our findings to other populations and ethnicities warrants further investigation.

## Conclusions

We first built the nomogram model for DLBCL patients, with satisfactory prediction and excellent discrimination, which might play an essential role in helping physicians enact better treatment strategies at the time of initial diagnosis. Age of diagnosis, gender, race, tumor grade, Ann Arbor stage, radiation, and chemotherapy were predictors for risk of CVM. Clinical variables at diagnosis can identify DLBCL patients at high risk of CVM, for whom preventive interventions should be considered.

## Data Availability

The data set analysis of this study can be found in SEER database. The datasets analyzed in this study are available in the SEER repository and can be obtained from: https://seer.cancer.gov/data/.

## Supplementary material

Supplementary Figure 1 Calibration curves of the nomogram of DLBCL patients for 5-, 10-, and 15-year CVM rates in the training cohort (**A**–**C**) and the internal validation cohort (**D**–**F**).
