# Supplementary figures and images for: Development and validation of a nomogram for predicting cardiovascular mortality risk for diffuse large B-cell lymphoma in children, adolescents, and adults
Source: Front Pediatr. 2024 Feb 7;12:1346006. doi: 10.3389/fped.2024.1346006 (PMC10879433; doi:10.3389/fped.2024.1346006)

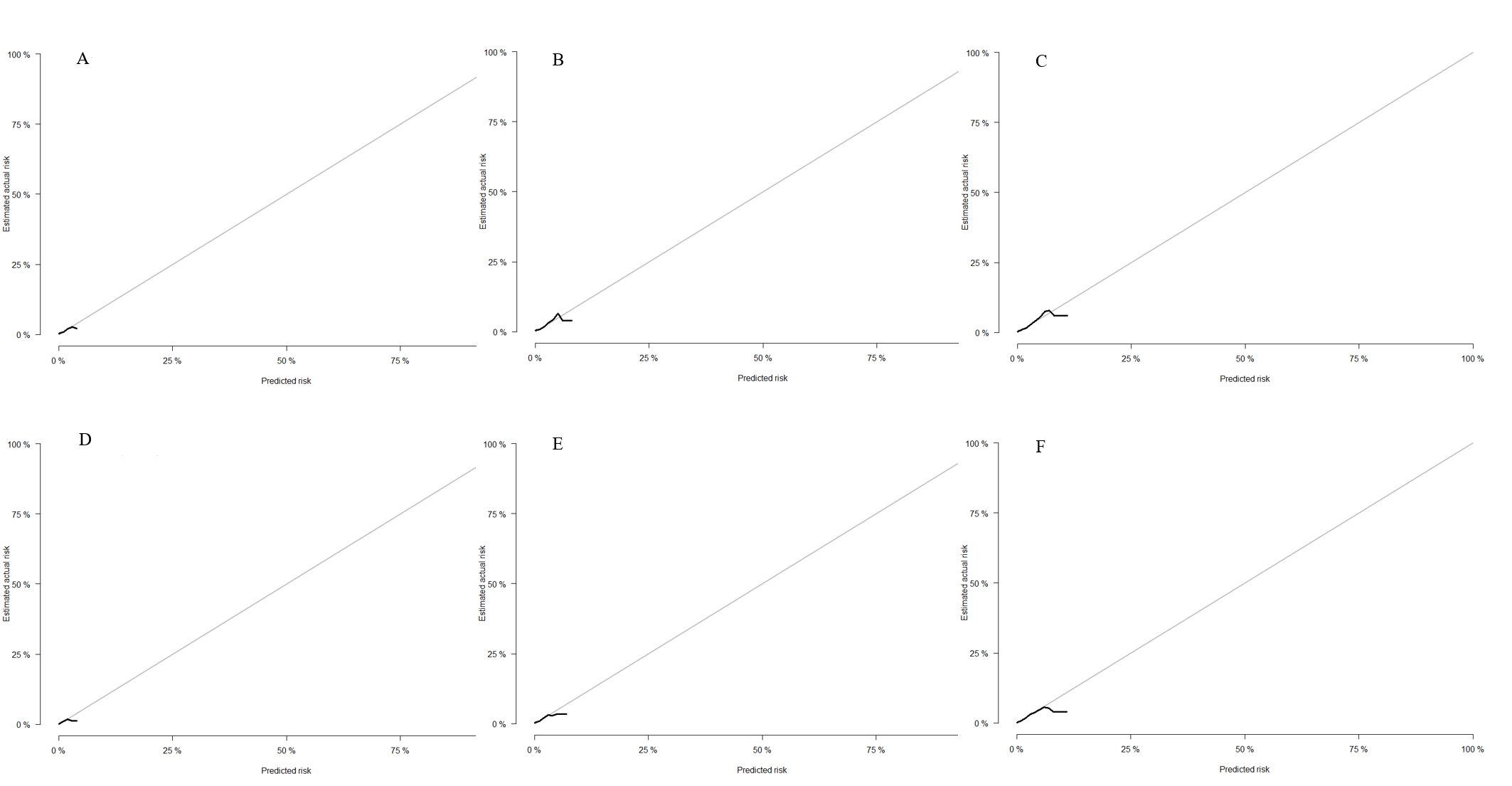

Supplement: Supplementary Figure 1 — Calibration curves of the nomogram of DLBCL patients for 5-, 10-, and 15-year CVM rates in the training cohort (A–C) and the internal validation cohort (D–F). [file Image1.tif]
